# COVID-19 Presenting as Encephalopathy in the Emergency Department: A Case Report

**DOI:** 10.5811/cpcem.2020.12.50036

**Published:** 2021-01-26

**Authors:** Travis B. Goodloe, Lauren A. Walter

**Affiliations:** University of Alabama Birmingham, Department of Emergency Medicine, Birmingham, Alabama

**Keywords:** COVID-19, encephalopathy, neuropathic, fever, case report

## Abstract

**Introduction:**

The severe acute respiratory syndrome coronavirus-2 (SARS-CoV-2), the etiology of the coronavirus disease 2019 (COVID-19) pandemic, has proven to be an era-defining illness with profound impact on patients and healthcare providers alike. By nearly all measures, daily cases and deaths are growing on a global scale despite conscious infection control efforts. As the medical community strives to better understand the pathogenesis of COVID-19, it has become increasingly appreciated that this “respiratory virus” can present clinically with a wide range of signs and symptoms not necessarily confined to the respiratory system.

**Case Report:**

Specifically, the central nervous system has been described as the presenting complaint of COVID-19, including anosmia and headaches and, more rarely, meningitis. This clinical case highlights the presentation of a 52-year-old male who presented to the emergency department with altered mental status and fever, ultimately attributed to COVID-19 infection.

**Conclusion:**

This case serves to add to the growing body of evidence surrounding the potentially severe neuropathologic capabilities of the novel SARS-CoV-2 virus.

## INTRODUCTION

Severe acute respiratory syndrome coronavirus-2 (SARS-CoV-2), known to cause coronavirus disease 2019 (COVID-19), is primarily considered a viral agent that causes respiratory disease; however, there is increasing evidence regarding the neuropathic capabilities and manifestations of the virus. Neurologic manifestations have been reported to range from mild accounts of headache, ageusia, and anosmia to more severe cases of encephalitis, myelitis, Guillain-Barré syndrome, polyradiculopathy, and even cerebrovascular disease.^1^, ^2^ It has been noted that patients with predisposing comorbidities such as hypertension, diabetes, and coronary artery disease are at increased risk for neurological sequelae of COVID-19.^3^

Neurologic involvement is thought to occur through several potential mechanisms. These include central nervous system access via specific receptors, namely the angiotensin converting enzyme 2 (ACE2) receptor as well as retrograde viral movement along axons, most frequently the olfactory nerve. It has also been suggested that neurological injury may be due to cytokine toxicity-related injury or secondary hypoxia due to cerebral edema and ischemia.^2^,^3^ Of these proposed mechanisms, the neurotoxicity related to cytokine storm has garnered significant attention. It is thought that the hypoxic and metabolic changes that occur due to the presence of cytokines in the brain parenchyma lead to dysregulation of typical metabolic processes.^4^, ^5^ This subsequently can present clinically as encephalopathy, which can be defined as diffuse brain dysfunction that manifests as varying degrees of altered consciousness.^4^ Our intent in this case report was to further contribute to the growing body of literature describing the neurological presentations and manifestations of COVID-19.

## CASE REPORT

A 52-year-old male with a past medical history of hypertension, diabetes mellitus type 2, end-stage renal disease (ESRD) on hemodialysis (HD), coronary artery disease status post coronary artery bypass grafting, and past cerebrovascular accident presented to the emergency department (ED) with altered mental status. The patient had been well, per family, including an uncomplicated HD session earlier that day, until the evening of presentation when family noted that he had increasing confusion and agitation, progressive for three to four hours, prompting them to call 911. On arrival to the patient’s home, paramedics noted that he was severely agitated, moaning loudly, and “jerking about,” particularly the bilateral upper extremities. To facilitate safe transfer to the hospital and due to paramedic concern for possible seizure-like activity, they administered midazolam five milligrams (mg) intramuscularly.

Upon presentation to the ED, the patient was noted to be febrile (38.94°C), tachycardic (115 beats per minute), and hypertensive (blood pressure 224/104 millimeters mercury (mmHg). He was moaning loudly with no coherent speech and unable to follow specific commands; however, he was responding to painful stimuli and appeared to respond to his name. Additionally, the patient was moving all extremities spontaneously and had no appreciable cranial nerve defects. The remainder of his physical exam revealed clear breath sounds bilaterally, no abdominal distension or perceived tenderness, and warm, dry, intact skin with no signs of breakdown or ulcers. Further evaluation and treatment were initiated, and the patient was started on vancomycin, ceftriaxone, azithromycin, and acyclovir for empiric meningitis coverage. A clevidipine infusion at one mg per hour was also started in the ED.

One hour after arrival and treatment initiation, the patient’s blood pressure was 143/90 mm Hg, and the infusion was discontinued. Additionally, chest radiograph did not show any acute abnormality, and computed tomography (CT) of the head without contrast showed chronic changes in the left putamen in the setting of known remote cerebrovascular accident. Laboratory evaluation was significant for an elevated blood urea nitrogen of 60 mg per deciliter (mg/dL) (reference range: 7–20 mg/dL) and creatinine of 8.3 mg/dL (0.8–1.2 mg/dL) in the setting of known ESRD. Otherwise, urinalysis did not show evidence of infection, urinary drug screen was negative, white blood cell count was 7.05 × 10^3^/microliter (mL) (4.5–11 × 10^3^/mL), and salicylate and acetaminophen levels were within normal limits. However, nasopharyngeal polymerase chain reaction (PCR) swab returned positive for SARS-CoV-2. A lumbar puncture was also performed, which evidenced clear cerebrospinal fluid (CSF), white blood cell count of zero per cubic millimeter (mm^3^) (0–5/mm^3^), protein of 46 mg/dL (10–60 mg/dL), and mildly elevated glucose of 121 mg/100 mL (50–80 mg/100 mL). The patient was admitted to the intensive care unit (ICU) for further management.

**CPC-EM Capsule**

What do we already know about this clinical entity?

Coronavirus disease 2019 (COVID-19) has primarily emerged as an upper airway and respiratory disease; however, it manifests in a myriad of clinical presentations.

What makes this presentation of disease reportable?

We detail a case of a 52-year-old male who presented with encephalopathy attributed to the virus but without any respiratory symptoms.

What is the major learning point?

Continued reporting of novel presentations of this disease entity will hone providers’ clinical acumen and ability to recognize COVID-19 in less typical, non-respiratory presentations.

How might this improve emergency medicine practice?

Continued research regarding viral pathology and the array of presentations will enable providers to identify and intervene with patients presenting with this deadly virus.

During ICU admission, the patient remained encephalopathic despite administration of antipyretics in the ED. His blood pressure remained stable with systolic less than 180 mm Hg with lisinopril 20 mg and amlodipine 10 mg daily. Antibiotic coverage was discontinued after approximately 48 hours as no signs of infection were present clinically or by laboratory evidence, and CSF culture returned negative (including varicella, herpes simplex, and cytomegalovirus). Magnetic resonance imaging (MRI) of the brain was performed on the morning of ICU day two, which did not show any acute pathologic changes. Continuous electroencephalogram for 24 hours was begun approximately six hours after initial presentation and showed evidence of mild, diffuse cerebral dysfunction. On ICU day three, the patient’s mentation began to improve, and he was noted to be increasingly alert, able to follow basic commands and speak simple phrases. On hospital day four, the patient was downgraded to a floor bed setting where he remained for an additional five days until his mentation was considered “back to baseline” with subsequent hospital discharge.

Repeat SARS-CoV-2 testing during outpatient follow-up 20 days after his initial ED presentation was negative. To date, the patient appears to have fully recovered from this episode of encephalopathy without neurologic sequelae.

## DISCUSSION

Medical literature that has emerged during the pandemic, including some case reports, has provided clinical anecdotes as well as evidence of how COVID-19 may present with neurological signs and symptoms. Although the specific mechanism for neurologic involvement is still being investigated, patients with complex medical histories and comorbidities appear to be at an increased risk for the neurological complications of SARS-CoV-2 infection.^6^

This case presentation of a 52-year-old male with multiple comorbidities who presented with febrile encephalopathy and SARS-CoV-2 positive serology in the absence of any other identifiable infectious or metabolic source provides an additional example of the neuropathologic capabilities of SARS-CoV-2. Although this patient’s CSF studies did not show findings consistent with inflammation, including pleocytosis or elevated protein, this is not an uncommon finding in viral encephalitis.^3^ Likewise, neurological imaging was unremarkable for acute processes; however, again, normal CT and/or MRI imaging is not uncommon in viral encephalitis, particularly early in the disease process.^3^ Posterior reversible encephalopathy syndrome was also considered but thought to be less likely in the absence of neuroradiographic abnormalities.^7^

Of note, the patient did present with significantly elevated blood pressure, which has been reported to be a major complicating factor in many SARS-CoV-2 cases.^8^ It is thought that as cerebral blood flow becomes compromised at increasingly elevated blood pressures, encephalopathy can develop due to the subsequent hypoxic effects.^9^ The virus’ effect on ACE2 receptors in the brain likely also plays a role in this dynamic, especially in patients already taking ACE inhibitors. There is no consensus at this time on the exact mechanisms involved, but viral binding to the ACE2 receptor may lead to an element of renin-angiotensin-aldosterone system disruption that could exacerbate or alter disease presentation in those with pre-existing hypertension.^10^

Additionally, it is important to note that encephalopathy is pathologically different from true encephalitis, which is characterized by inflammation of the brain itself. Reported cases of SARS-CoV-2 presence in CSF as evidenced by pleocytosis and PCR detection, as well as acute changes visualized on neurological imaging, exist in the literature.^11^ However, literature review reveals that the predominance of reported encephalopathic presentations of patients infected with SARS-CoV-2 are not found to have these aforementioned features present.^3^ Because neurological manifestations of SARS-CoV-2 are being increasingly recognized, clinical consideration of infection with this virus cannot be excluded in patients who present to the ED with new-onset neurological complaints or symptoms. It has been clearly established that these symptoms alone may be the only initial features of a SARS-CoV-2 positive patient’s clinical picture.

Lastly, it is of note that if neurological involvement is ultimately found to be primarily mediated by immune dysfunction and cytokine storm as discussed previously, then this greatly expands the potential for treatment of COVID-19 through utilization of existing drug agents that function to dampen this response. In this way, these treatment modalities may have utility in this unique subset of patients presenting with significant neurological manifestations.

## CONCLUSION

The coronavirus disease 2019 has undoubtedly raised significant questions and prompted a vast mobilization of research due to its novelty and its increasingly recognized variability in clinical presentation. The case presented in this report is evidence of this variability and underscores the importance of considering COVID-19 as a potential cause of a wide range of neurological manifestations. In today’s healthcare environment, providers should not overlook COVID-19 as a source of altered mental status, especially in the context of patients presenting to the ED. Without question, further research and evaluation is needed to better understand the neuropathologic capacities of COVID-19 as well as its long-term impacts on those with pre-existing medical comorbidities.
